# Altered Intracellular Milieu of ADAR2-Deficient Motor Neurons in Amyotrophic Lateral Sclerosis

**DOI:** 10.3390/genes8020060

**Published:** 2017-02-08

**Authors:** Takenari Yamashita, Megumi Akamatsu, Shin Kwak

**Affiliations:** 1 Graduate School of Medicine, University of Tokyo, Bunkyo-ku, Tokyo 113-0033, Japan; yamashitat-tky@umin.net (T.Y.); makamatsu@m.u-tokyo.ac.jp (M.A.); 2 Clinical Research Center for Medicine, International University of Health and Welfare, Ichikawa, Chiba 272-0827, Japan

**Keywords:** amyotrophic lateral sclerosis (ALS), adenosine deaminase acting on RNA 2 (ADAR2), RNA editing, transactive response DNA-binding protein (TDP-43), Ca^2+^-permeability

## Abstract

Transactive response DNA-binding protein (TDP-43) pathology, and failure of A-to-I conversion (RNA editing) at the glutamine/arginine (Q/R) site of α-amino-3-hydroxy-5-methyl-4-isoxazole propionic acid (AMPA) receptor subunit GluA2, are etiology-linked molecular abnormalities that concomitantly occur in the motor neurons of most patients with amyotrophic lateral sclerosis (ALS). Adenosine deaminase acting on RNA 2 (ADAR2) specifically catalyzes GluA2 Q/R site-RNA editing. Furthermore, conditional ADAR2 knockout mice (AR2) exhibit a progressive ALS phenotype with TDP-43 pathology in the motor neurons, which is the most reliable pathological marker of ALS. Therefore, the evidence indicates that ADAR2 downregulation is a causative factor in ALS, and AR2 mice exhibit causative molecular changes that occur in ALS. We discuss the contributors to ADAR2 downregulation and TDP-43 pathology in AR2 mouse motor neurons. We describe mechanisms of exaggerated Ca^2+^ influx amelioration via AMPA receptors, which is neuroprotective in ADAR2-deficient motor neurons with normalization of TDP-43 pathology in AR2 mice. Development of drugs to treat diseases requires appropriate animal models and a sensitive method of evaluating efficacy. Therefore, normalization of disrupted intracellular environments resulting from ADAR2 downregulation may be a therapeutic target for ALS. We discuss the development of targeted therapy for ALS using the AR2 mouse model.

## 1. Introduction

Amyotrophic lateral sclerosis (ALS) is the most common adult-onset motor neuron disease, and is characterized by progressive loss of both upper and lower motor neurons. The disease course is rapid and respiratory muscle weakness results in death within a few years following onset. More than 90% of ALS cases are sporadic, in which the cause of ALS has not been determined. The remaining cases of ALS are familial, and approximately half of these cases are associated with gene mutations, including at superoxide dismutase 1 (*SOD1*), chromosome 9 open reading frame 72 (*C9ORF72*), *TARDBP* (the gene encoding transactive response DNA-binding protein or TDP-43), and fused in sarcoma (*FUS*) [1]. Varied disease mechanisms leading to motor neuron death in ALS include RNA misprocessing (metabolism), protein misfolding, and excitotoxicity [2,3]. Furthermore, therapies that effectively alter the disease course are not currently available.

In sporadic ALS and some familial ALS, including *C9ORF72*- and *TARDBP*-associated ALS, loss of transactive response element DNA/RNA binding protein 43 (TDP-43) in the nucleus and abnormal TDP-43 positive cytoplasmic inclusions (TDP-43 pathology) in motor neurons are observed [3,4,5,6]. TDP-43 is a nuclear protein involved in regulating RNA processing and protein misfolding [7,8,9,10]; however, the molecular mechanisms of TDP-43 pathology are not well-understood. Additionally, dominant mutations in the *TARDBP* gene, which encodes this protein, have been identified in patients with ALS, and there is evidence that these mutations are responsible for the pathogenesis of ALS [11]. TDP-43 pathology is a pathological hallmark of ALS because it is observed in the motor neurons of most patients with ALS.

**Figure 1:**
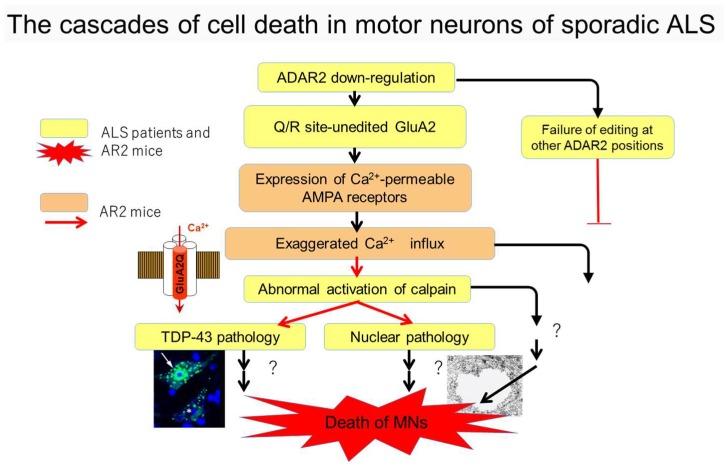
Cell death cascade of motor neurons in sporadic amyotrophic lateral sclerosis (ALS). Molecular abnormalities relevant to neuronal death only in conditional adenosine deaminase acting on RNA 2 (ADAR2) knock-out mice (AR2) (orange), as well as in both patients with ALS and AR2 mice (yellow) have been experimentally validated. AMPA = α-amino-3-hydroxy-5-methyl-4-isoxazole propionic acid; TDP-43 = transactive response DNA-binding protein; MNs = motor neurons.

Another molecular abnormality is that excitatory neuron death occurs via α-amino-3-hydroxy-5-methyl-4-isoxazole propionic acid (AMPA) receptors in ALS [12]. RNA editing failure at the glutamine/arginine (Q/R) site of the GluA2 AMPA receptor subunit is observed in the motor neurons of most patients with sporadic ALS [13,14,15]. Adenosine deaminase acting on RNA 2 (ADAR2), the enzyme specifically responsible for RNA editing at the Q/R site of GluA2 [16,17], is downregulated in the motor neurons of patients with ALS [13,18]. TDP-43 pathology and failure of RNA editing are observed in the motor neurons of patients with sporadic ALS; therefore, both factors may be disease-specific abnormalities. Furthermore, TDP-43 pathology, including insoluble, hyperphosphorylated, and aggregation-prone TDP-43 fragments, was exclusively observed in motor neurons with reduced ADAR2 activity in patients with sporadic ALS [17,18], suggesting a molecular link between these events. Moreover, analyses of conditional ADAR2 knockout mice (ADAR2*^flox^*^/*flox*^/VChAT-Cre.Fast; AR2 mice) demonstrated that insufficient ADAR2 expression induced motor neuron death via an abnormal Ca^2+^-permeable AMPA receptor-mediated mechanism [19]. Notably, AR2 mice exhibit mislocalization of TDP-43 from the nucleus to the cytoplasm in ADAR2-lacking motor neurons [20]. The pathogenesis of ADAR2 and TDP-43 containing phosphorylated TDP-43 or other patterns of TDP-43 pathology in sporadic ALS is similar to that observed in AR2 mice [18,19,20,21,22]. Figure 1 depicts a pathogenesis cascade for the mechanism of Ca^2+^ upregulation via influx through AMPA receptors, including expression of Q/R site-edited GluA2 in the absence of ADAR2 activating calpain, which generates TDP-43 pathology or induces motor neuron death. Therefore, normalization of disrupted intracellular environments resulting from ADAR2 downregulation may be a therapeutic target for ALS treatments. The present review addresses the development of targeted therapy for ALS using the AR2 mouse as a mechanistic model.

## 2. ADAR2 in Sporadic ALS

**Figure 2:**
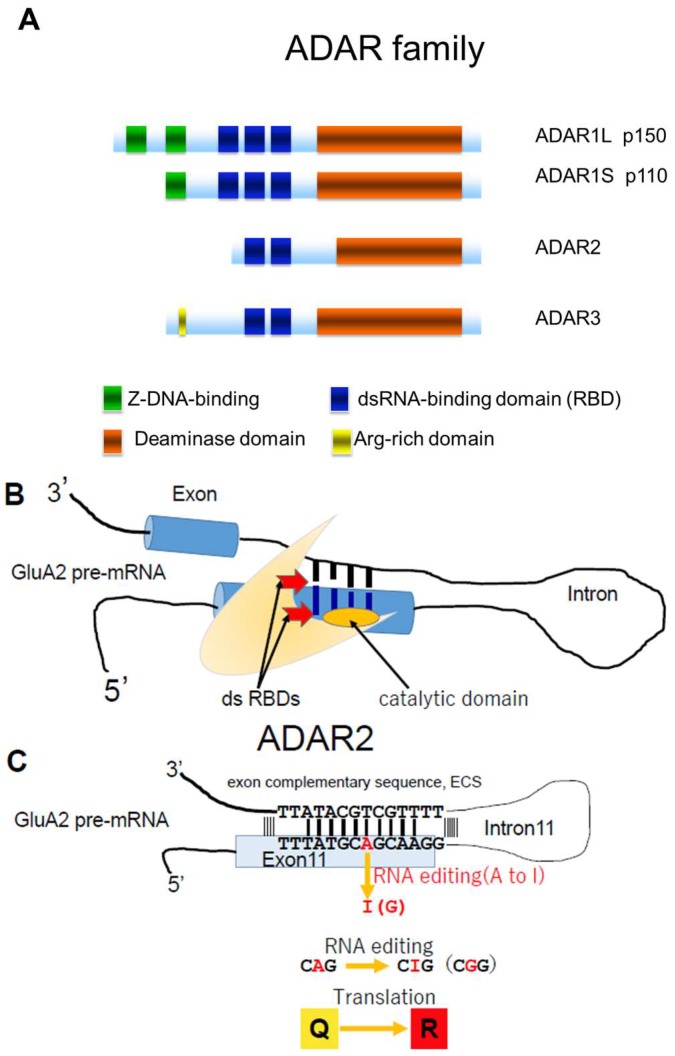
Adenosine deaminase acting on RNA (ADAR) structure and RNA editing. (**A**) Three members of the ADAR family. ADAR1 has two isoforms (p150 and p110). ADAR1p110 exists in the nucleus and ADAR1p150 exists in both the nucleus and the cytoplasm. ADAR2 is primarily localized in the nucleus of brain and spinal cord and catalyzes A-to-I conversion at the GluA2 glutamine/arginine (Q/R) site. Z-DNA-binding domain (green), RBD; double strand RNA (dsRNA) binding domain (blue), deaminase domain (orange), and arginine-rich domain (yellow); (**B**) ADAR2 has a double-stranded (ds) RNA binding domain (RBD; red) and a deaminase domain (orange); (**C**) dsRNA is formed at the distal end of exon 11, which includes the coding sequence for the Q/R site and the exon complementary sequence (ECS) in intron 11 of GluA2 pre-mRNA. ADAR2 catalyzes conversion of adenosine to inosine (A-to-I RNA editing) in the dsRNA structure in both coding and non-coding regions of various transcripts. The adenosine at the Q/R site of GluA2 pre-mRNA is converted to inosine (A-to-I conversion), the genomic CAG (codon for Q) is converted to CIG in mRNA, and CIG is read as CGG, which is a codon for R during translation.

The RNA editing enzyme ADAR, possesses two or three double-stranded RNA (dsRNA) binding domains in the N-terminal region, as well as one deaminase domain in the C-terminal region [23,24] (Figure 2A). ADARs convert adenosine (A) to inosine (I) in dsRNA. In mammals, including humans, three structurally-related ADAR family members have been identified (ADAR1, ADAR2, and ADAR3) (Figure 2A). ADAR1 and ADAR2 actively catalyze A-to-I conversion in many tissues, whereas ADAR3 is predominantly expressed in brain white matter and exhibits no known editing activity [25,26]. More than two million A-to-I positions have been identified in both coding and noncoding regions of numerous gene transcripts [24,27,28,29]. Changes in ADAR activity at many A-to-I positions are associated with a wide spectrum of human diseases, including cancers, neurological disorders, metabolic diseases, viral infections, autoimmune disorders, and cocaine addiction [24,30]. Expression of ADAR2, but not ADAR1 or ADAR3, significantly decreases in motor neurons of patients with sporadic ALS compared to healthy control participants [13]. This inefficient expression of ADAR2 results in expression of Q/R site-unedited GluA2, which results in AMPA receptors being abnormally permeable to Ca^2+^. Here, we review evidence indicating that decreased ADAR2 activity in motor neurons initiates a cell death cascade, which is relevant to the pathogenesis of sporadic ALS (Figure 1).

## 3. Q/R Site-Editing of AMPA Receptor Subunit GluA2 and Ca^2+^-Permeability of the AMPA Receptors in Sporadic ALS

**Figure 3:**
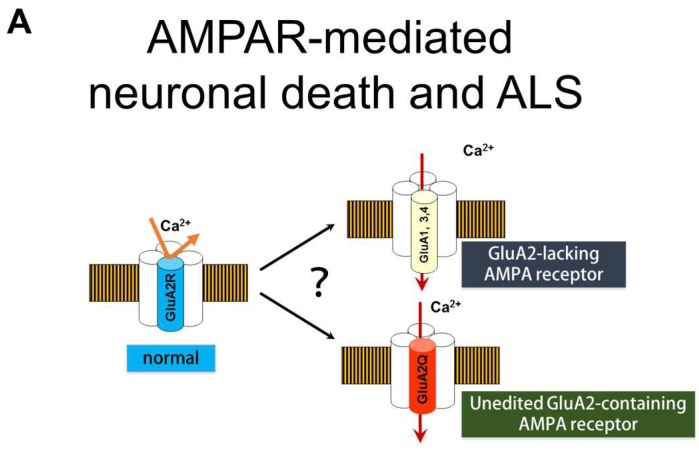

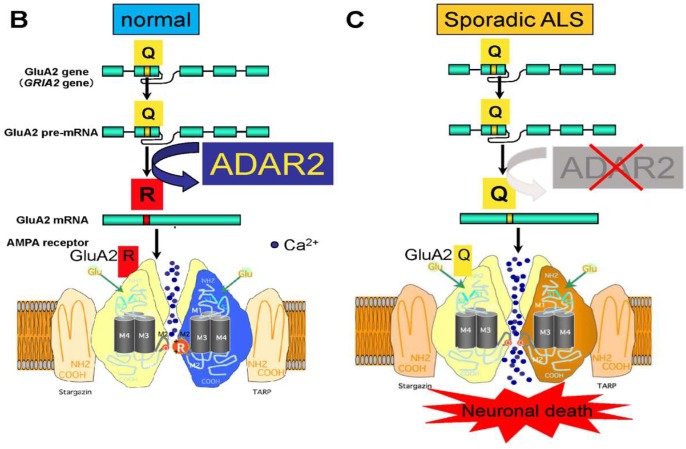
The α-amino-3-hydroxy-5-methyl-4-isoxazole propionic acid (AMPA) receptor and Ca^2+^-permeability. (**A**) AMPA receptors consist of homo- or hetero-tetramers of four subunits (GluA1 to GluA4). Ca^2+^-impermeable AMPA receptors contain a GluA2 subunit (edited GluA2 at the Q/R site; GluA2R, blue), whereas physiologically expressed Ca^2+^-permeable AMPA receptors do not contain a GluA2 subunit and consist of GluA1, GluA3, and GluA4 subunits (yellow). In sporadic amyotrophic lateral sclerosis (ALS), Ca^2+^-permeable AMPA receptors containing unedited GluA2 (GluA2Q, red) are expressed in motor neurons; (**B**,**C**) The diagram shows how abnormally Ca^2+^-permeable AMPA receptors are expressed in motor neurons of patients with sporadic amyotrophic lateral sclerosis (ALS); (**B**) In healthy motor neurons, the GluA2 Q/R site is edited by adenosine deaminase acting on RNA 2 (ADAR2), and AMPA receptors containing GluA2R (blue) are Ca^2+^-impermeable. (**C**) In motor neurons of patients with sporadic ALS, the GluA2 Q/R site is unedited, due to deficient ADAR2 expression. Therefore, AMPA receptors containing GluA2Q (yellow) are Ca^2+^-permeable, which induces neuronal death via exaggerated Ca^2+^ influx.

AMPA receptors are a subtype of ionotropic glutamate receptors, and are composed of homo- or hetero-tetramers of four GluA subunits (GluA1 to GluA4) (Figure 3A) [31]. The Ca^2+^-permeability of AMPA receptors is determined by the presence or absence of the GluA2 subunit; Ca^2+^-impermeable AMPA receptors contain at least one GluA2 subunit, whereas Ca^2+^-permeable AMPA receptors are comprised of GluA1, GluA3, and/or GluA4 subunits only, and lack GluA2 (Figure 3A) [32]. The GluAs have four membrane domains (M1 to M4) (Figure 3) and the glutamine/arginine (Q/R) site is located in the M2 domain that faces the channel pore of the AMPA receptor. Although all GluA genes (*Gria1* to *Gria4*) have a CAG sequence (a codon for Q) at the Q/R site, GluA2 but not the other three GluAs, has an R at the Q/R site (Figure 3). This Q to R conversion in the GluA2 protein results from A-to-I RNA editing at the Q/R site, which is mediated by ADAR2; a CAG sequence (a codon for Q) at the Q/R site is converted to CIG in the pre-mRNA, and CIG is read as CGG during translation, which is a codon for R. A-to-I RNA editing occurs only in the pre-mRNA of GluA2, but not in that of other GluAs, because the distal end of exon 11 contains the Q/R site, which forms double strand sequences with the exon complementary sequence (ECS) in intron 11 of GluA2 pre-mRNA. ADARs act on dsRNAs, and of the AMPA receptor subunit genes, only *Gria2* possesses an ECS in intron 11, therefore, A-to-I conversion occurs only in GluA2 pre-mRNA [16]. Moreover, neurons express only Q/R site-edited GluA2 because A-to-I conversion at the Q/R site occurs for all GluA2 mRNA [14,15,33]. Therefore, the large, positively charged R residue at the Q/R site prevents Ca^2+^ passing through the channel pore of the AMPA receptor (Figure 3B) [34].

In the spinal motor neurons of most patients with sporadic ALS, RNA editing of GluA2 is inefficient at the Q/R site, and abnormal Q/R site-unedited GluA2 (GluA2Q) is expressed in approximately half of the patients’ remaining motor neurons [13,14,15]. AMPA receptors are either Ca^2+^-permeable or -impermeable; this is determined based on whether the AMPA receptor has GluA2R in its subunit assembly. Only the AMPA receptors with GluA2R are Ca^2+^-impermeable, whereas those composed of only Q/R site-unedited subunits, including GluA2Q, are Ca^2+^-permeable [34]. When unedited GluA2 (GluA2Q) is expressed in the motor neurons, AMPA receptors are abnormally Ca^2+^-permeable increasing motor neurons death in conditional ADAR2 knockout mice (AR2) (Figure 3C) [22].

## 4. ADAR2 Downregulation Results in Motor Neuron Death in AR2 Mice

In order to assess whether the expression of GluA2Q resulting from ADAR2 downregulation is a cause of neuronal death, we developed a conditional ADAR2 knockout mouse line using a Cre-loxP system (ADAR2*^flox^*^/*flox*^/VAChT-Cre.Fast) [19]. AR2 mice exhibited progressive declines in motor function, in parallel with progressive loss of ADAR2-deficient motor neurons. ADAR2-deficient motor neurons, which accounted for approximately 50% of motor neurons, expressed only Q/R site-unedited GluA2. The remaining ADAR2-expressing motor neurons expressed only Q/R site-edited GluA2 in AR2 mice. ADAR2-deficient motor neuron death was rescued by the expression of Q/R site-edited GluA2, in the absence of ADAR2, in AR2-rescued (AR2res) mice (ADAR2*^flox^*^/*flox*^/VAChT-Cre.Fast/GluR-B^R/R^), indicating that expression of Q/R site-unedited GluA2 may cause motor neuron death. Motor neuron death was slow, but virtually all ADAR2-deficient motor neurons disappear by one year of age. The ALS-like phenotypes of AR2 mice suggest a pathogenic role of the failure of GluA2 Q/R site RNA editing, but not that of other ADAR2-mediated RNA editing sites in ALS [19,21]. However, fragmentation, mutation, and mislocalization of TDP-43 did not alter ADAR2 expression [35]. Together, the results indicated that abnormal TDP-43 processing is not an upstream event of inefficient GluA2 Q/R site RNA editing in the motor neurons of patients with sporadic ALS.

## 5. Cell Death Cascades in ALS

Abnormal processing and mislocalization of TDP-43 in motor neurons is the most reliable pathological indicator of sporadic ALS and some types of familial ALS, including *C9ORF72*-associated ALS [3,4,5]. TDP-43 is a nuclear protein involved in transcript regulation and alternative splicing [8,9]. TDP-43 pathology consists of mislocalization of TDP-43 from the nucleus to the cytoplasm, and formation of abnormal cytoplasmic inclusions. In addition, TDP-43 is abnormally fragmented and phosphorylated in pathology-affected tissues [4,5,36,37]. Additionally, TDP-43 pathology is not specific to ALS motor neurons, and frontotemporal lobular degeneration-TDP occurs in cortical neurons. This is also observed in various neurological diseases, including Alzheimer′s disease, traumatic brain injury, hippocampal sclerosis, Lewy body dementia, Pick′s disease, corticobasal degeneration, agryrophilic gain disease, and Huntington’s disease [6,38,39,40,41,42,43,44,45,46].

Both TDP-43 pathology and deficient RNA editing at the GluA2 Q/R site are molecular abnormalities specifically associated with ALS; therefore, a molecular link between TDP-43 pathology and deficient RNA editing was investigated. When the spinal cords of patients with sporadic ALS were examined using immunohistochemistry, TDP-43 pathology was always observed in the motor neurons without ADAR2 immunoreactivity. Conversely, all motor neurons lacking TDP-43 pathology were positive for ADAR2 immunoreactivity [18].

**Figure 4:**
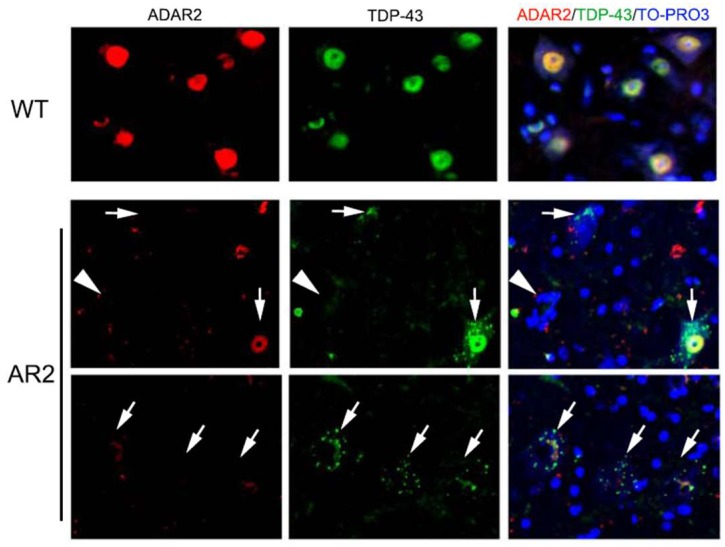
Transactive response DNA-binding protein (TDP-43) mislocalization and adenosine deaminase acting on RNA 2 (ADAR2) in anterior horn cells (AHCs) of spinal cord. Immunofluorescence assays revealed that ADAR2 (red) and TDP-43 (green) are both expressed in the nucleus of AHCs in wild type mice. TDP-43 immunoreactivity was absent in the AHCs of ADAR2 knockout mice (AR2; arrowhead). AHCs with low or no immunoreactivity to ADAR2 showed TDP-43 immunoreactivity in the cytoplasm and TDP-43-positive cytoplasmic aggregates in AR2 mice (arrow). TO-PRO-3 is a cellular maker (blue).

The immunohistochemical observations prompted investigations of the mechanisms of TDP-43 pathology. A subsequent study demonstrated that homozygous (AR2) and heterozygous (AR2H) conditional ADAR2 knockout mice [19] exhibited mislocalization of TDP-43 from the nucleus to the cytoplasmic aggregates, resembling the TDP-43 pathology in ADAR2-deficient motor neurons (Figure 4) [20]. Furthermore, an in vitro calpain assay demonstrated that TDP-43 was specifically cleaved by calpain, a Ca^2+^-dependent cysteine protease. The fragments of TDP-43 in the in vitro calpain assay and in tissues from AR2 mice exhibited a similar pattern of molecular bands, as demonstrated by Western blotting. The role of calpain was further supported by normalization of subcellular TDP-43 localization in AR2res mouse motor neurons for which Q/R site-edited GluA2 was expressed in the absence of ADAR2, which demonstrated normal Ca^2+^ influx through AMPA receptors. These results indicate that exaggerated Ca^2+^ influx through AMPA receptors, resulting from failure of the GluA2 Q/R site RNA editing, activated calpain and resultant calpain-dependent TDP-43 fragments, thereby contributing to formation of cytoplasmic inclusions [20]. Furthermore, calpain-dependent N-terminal TDP-43 fragments were aggregation-prone. Additionally, the activated form of calpain is more abundant in the brain and spinal cord tissue from patients with ALS than in healthy control participants. Calpain-dependent TDP-43 fragments occur in the lysates and sarkosyl-insoluble fractions of patient tissues [20]. These results suggest that calpain-dependent cleavage of TDP-43 is likely involved in the pathogenesis of TDP-43 pathology in ALS motor neurons, similarly to observations in AR2 mouse motor neurons. Excessive calpain activity cleaves TDP-43 into soluble fractions; therefore, cytoplasmic aggregates of TDP-43 are more abundant in AR2H mice than in AR2 mice. This finding suggests that a moderate increase in the Ca^2+^ influx creates an optimal intracellular milieu for TDP-43 pathology in ALS motor neurons. Together, these results suggest that the Ca^2+^-permeable AMPA receptor-mediated neuronal cell death mechanism that occurs in AR2 mice also occurs in the motor neurons of patients with ALS. Therefore, we propose a neuronal death cascade in both patients with ALS and AR2 mice, which is illustrated in Figure 1.

ADAR2-deficient motor neurons die in AR2 mice and these neurons exhibit TDP-43 mislocalization, therefore, it is likely that reduced motor neuron death is associated with normalization of TDP-43 subcellular localization. *ADAR2* gene delivery using adeno-associated virus (AAV) vectors [47] or oral administration of an AMPA receptor antagonist [48] upregulated both ADAR2 expression and GluA2 Q/R site RNA editing, thereby decreasing motor neuron death and TDP-43 mislocalization in AR2 mice. These results strongly support a role of Ca^2+^-permeable AMPA receptor-mediated mechanisms in sporadic ALS.

## 6. Subcellular Localization of TDP-43 in the ChAT Positive Anterior Horn Cells (AHCs) as Biomarkers of ALS Pathology

ALS is characterized by the progressive loss of both upper and lower motor neurons, causing progressive muscle weakness and atrophy. Over 30 genes that relate to familial occurrence of ALS have been identified [49]; however, most ALS cases are sporadic (no other family member has ALS) and few patients have any of the currently identified ALS-linked genes [2]. The most reliable pathological indicator of ALS other than loss of lower motor neurons is TDP-43 pathology in the remaining motor neurons, which is observed in most patients with sporadic ALS. However, most of the animal models that have been generated by expressing ALS-linked genes, including SOD1, the most widely used ALS model, do not exhibit TDP-43 pathology in motor neurons [50,51,52]; therefore, there are concerns regarding the validity of animal models of ALS.

As described above, homozygous AR2 and heterozygous AR2H mice exhibit mislocalization of TDP-43 in motor neurons. In AR2H mice, motor neurons with ablation of one *ADAR2* gene allele expressed unedited GluA2 mRNA in a maximum of 30% of all GluA2 mRNA, and approximately 20% of motor neurons underwent progressive and slow death [19,21]. Furthermore, ADAR2-deficient motor neurons from AR2 mice were devoid of TDP-43 immunoreactivity during the early post-natal period, whereas those from AR2H mice exhibited numerous TDP-43-positive cytoplasmic inclusions. Furthermore, some motor neurons from AR2H mice did not express nuclear TDP-43 immunoreactivity, similarly to observations in motor neurons of patients with ALS. The difference in TDP-43 immunoreactivity between AR2 and AR2H mice depends on differences in Ca^2+^ influx through the AMPA receptors, including those with unedited GluA2 [19,20,21]. The proportion of unedited GluA2 in the motor neurons of patients with ALS patients varied from 0% to 100%; therefore, lower Ca^2+^ influx through the AMPA receptors in AR2H mouse motor neurons represents human ALS motor neurons more closely than AR2 mouse motor neurons. These findings suggest that differences in subcellular localization of TDP-43 reflect the extent of Ca^2+^ influx through AMPA receptors in AR2 and AR2H mice. Furthermore, exaggerated Ca^2+^ influx through AMPA receptors causes motor neuron death in AR2 and AR2H mice. Therefore, subcellular localization of TDP-43 may differentiate motor neurons that will die from those that will survive. This property may be useful for assessing the therapeutic efficacy of ALS treatments.

The death cascade in AR2 mice that begins with ADAR2 downregulation likely contributes to motor neuron death in patients with ALS, therefore, we attempted to rescue motor neurons by restoring ADAR2 activity and by inhibiting the exaggerated Ca^2+^ influx through AMPA receptors [47,48]. In order to restore ADAR2 activity in non-ADAR2-expressing motor neurons, we delivered human ADAR2 cDNA to motor neurons using an adeno-associated virus serotype 9 (AAV9) vector. For ALS therapy, we needed to deliver the *ADAR2* gene to lower motor neurons that are located in the brain stem cranial motor nerve nuclei and the ventral horn of the spinal cord, therefore systemic administration was indicated. However, systemically applied molecules would not readily be transported across the blood-brain barrier (BBB) into the central nervous system. Therefore, AAV9 vectors were modified to facilitate crossing the BBB [47,53]. *ADAR2* gene delivery to ADAR2-deficient motor neurons at a level required to edit the GluA2 Q/R site was predicted to reinstate the ALS phenotype observed in AR2 mice. We injected the AAV9-ADAR2 vector into the tail vein of AR2 mice and evaluated the effects on progressive motor dysfunction and motor neurons death.

AMPA receptor antagonists are a potential new class of drug for neurological diseases, including ALS, but adverse effects have prevented their clinical use [54]. Recently, the non-competitive AMPA receptor antagonist, perampanel, has been approved as an anti-epileptic drug [55]. We tested the therapeutic effects of perampanel in AR2 mice during 90 days of oral administration.

**Figure 5:**
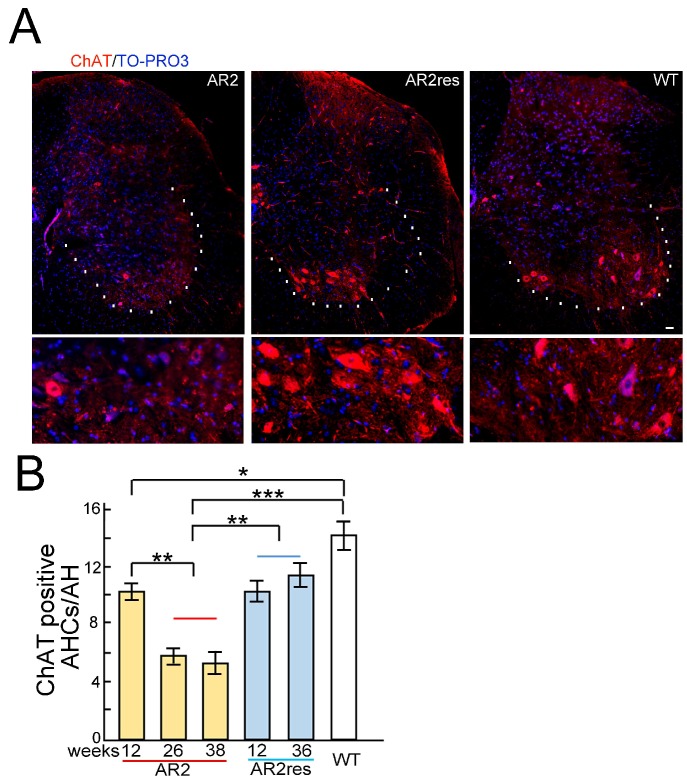
Choline acetyltransferase (ChAT) immunohistochemistry in the spinal cord of conditional adenosine deaminase acting on RNA 2 (ADAR2) knock-out mice (AR2) and AR2-rescued (AR2res) mice. (**A**) ChAT immunostaining (red) of the lumbar spinal cord. White dotted lines indicate the margin of the ventral gray matter. The bottom panels are the enlarged view of the upper panels. TO-PRO-3 (blue) was used as a cell marker. The scale bar indicates 20 µm; (**B**) As previously reported [19], the number of ChAT-positive anterior horn cells (AHCs) age-dependently decreases in AR2 mice compared to healthy controls. The age-dependent reduction in the number of ChAT-positive AHCs was rescued in AR2res mice. Means (columns) and standard errors of the means (SEMs; bars) are indicated. *n* = 5; * *p* < 0.05, ** *p* < 0.01, *** *p* < 0.001, Mann-Whitney U test against the value for wild type (WT) or AR2res groups.

**Figure 6:**
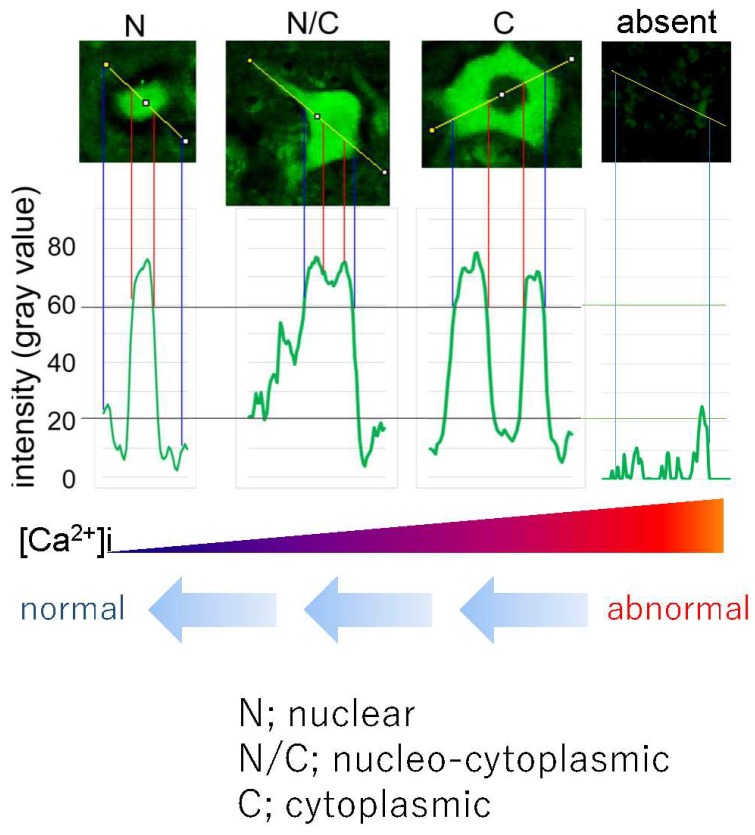
Subcellular localization of transactive response DNA-binding protein (TDP-43) and Ca^2+^ influx in adenosine deaminase acting on RNA 2 (ADAR2)-deficient motor neurons. Representative anterior horn cells (AHCs) with different TDP-43 subcellular localization patterns in the conditional ADAR2 knockout mice (AR2): predominantly nuclear (N), nucleo-cytoplasmic (N/C), cytoplasmic (C) or lack of immunoreactivity. The vertical axis indicates the intensity (gray value), which was evaluated with ImageJ software. The threshold for TDP-43 positivity was set at a level three-fold higher (60 gray) than the background intensity (20 gray). This figure is modified from a previously published figure [48].

The vector-based and antagonist treatments prevented progression of the ALS phenotype, including motor dysfunction, as assessed by rotarod retention time and grip strength [48]. More AHCs were also observed in the treated groups than in the untreated groups. Furthermore, the number of TDP-43-positive AHCs significantly increased in the treated groups compared to the untreated groups. When subcellular distribution patterns of TDP-43 were compared between the treated groups and untreated groups, the number of AHCs with nuclear TDP-43 immunoreactivity was significantly increased and those without TDP-43 immunoreactivity decreased in the treated groups (Figure 6) [48]. TDP-43 normally localizes in the nucleus; however, mislocalization from the nucleus to the cytoplasm may occur via cleavage by the Ca^2+^ dependent protease, calpain. Exaggerated Ca^2+^ influx through AMPA receptors activates calpain in the cytoplasm, and different subcellular distribution patterns of TDP-43 reflect varying cytoplasmic Ca^2+^ concentrations. Therefore, an increase in the proportion of motor neurons that exhibit nuclear TDP-43 localization may be a useful biomarker for evaluating ALS therapies (Figure 5 and Figure 6) [48].

**Figure 7:**
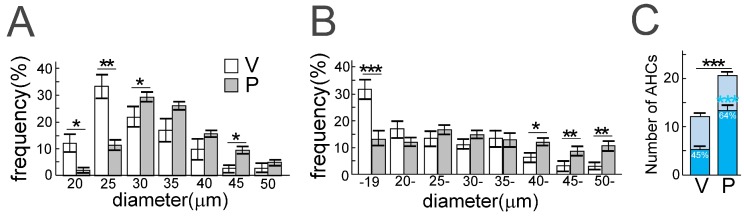
Perampanel administration for 90 days rescued anterior horn cells (AHCs) from death in conditional adenosine deaminase acting on RNA 2 (ADAR2) knockout mice (AR2). (**A**,**B**) Frequency histogram of AHC diameters in the perampanel group (P, gray columns) and the vehicle group (V, white columns): by TO-PRO-3 staining (**A**) and by choline acetyltransferase (ChAT) immunostaining (**B**). The vertical axis indicates the proportion of the total number of AHCs with diameters in each range; (**C**) The number of ChAT-positive AHCs with a diameter of more than 20 µm in AR2 mice treated with (P) or without (V) perampanel. V, vehicle (methyl cellulose)-treated AR2 mice (*n* = 5); P, perampanel-treated AR2 mice (*n* = 5). Means (columns) and standard errors of the mean (SEMs) (bars) are indicated. *n* = 5; * *p* < 0.05, ** *p* < 0.01, *** *p* < 0.001, Mann–Whitney U test against the value of the vehicle group. This figure is modified from a previously published figure [48].

Reduced cellular size should also be considered because this characteristic of motor neurons in patients with ALS predicts degeneration and atrophy. A frequency histogram of the diameter of AHCs in a perampanel group and a vehicle control group, using TO-PRO-3 staining and ChAT immunostaining, found that the diameter of ChAT-positive AHCs was significantly larger in the perampanel group compared to the control group (Figure 7). ChAT is a general marker for motor neurons; therefore, the findings indicate that the cellular size of motor neurons decreased with progression of ALS pathology in AR2 mice. Reductions in the number of ChAT-positive AHCs in AR2 mice were age-dependent, however, the number of ChAT-positive AHCs did not change in AR2res mice (Figure 5B). The finding that ADAR2-lacking motor neurons decrease in size before death is similar in both AR2 mice and patients with ALS [20,47,48].

## 7. Mechanisms of TDP-43 Mislocalization in Relation to the Motor Neuron Death Cascade

The mechanisms for the contributions of TDP-43 mislocalization to motor neuron death, as well as whether mislocalization of TDP-43 occurs as a component of the death cascade or whether it is merely an associated phenomenon that is observed downstream of ADAR2 downregulation, are key components of ALS pathology. Research examining death cascades observed downstream of ADAR2 downregulation found that the motor neurons of AR2 mice possess abnormal nuclear vacuoles, and that these vacuoles disappear when Ca^2+^ influx through AMPA receptors is normalized [56]. These results indicated the motor neurons of AR2 mice possess abnormal nuclear vacuoles during the course of death. Both nuclear volume and the RNA contents of motor neurons are decreased in patients with ALS compared to healthy control participants [57]. Over the last decade, abnormal RNA/DNA binding proteins, such as FUS/TLS and TDP-43, have been found to cause RNA processing abnormalities and functional defects in the nuclei of motor neurons in ALS [1,37,58,59]. In addition, pathological tissues of patients with ALS carrying the *C9ORF72* gene with hexanucleotide repeat expansions exhibit abnormal RNA foci and degeneration of nuclear function by nucleo-cytoplasmic transport [60,61,62,63]. Donnelly et al. demonstrated that *ADARB2* (ADAR3) interacts with G_4_C_2_ repeats in induced pluripotent stem cell (iPSC) neurons from patients with *C9ORF72* ALS [64]. ADAR3, however, has no editing activity, including at the GluA2 Q/R site [26]. Therefore, the mechanisms of hyperactivity in iPSC neurons with expanded G_4_C_2_ repeats remain unknown. Moreover, failure of GluA2 Q/R site-editing has recently been demonstrated in the motor neurons of patients with ALS who have the *FUS^P525L^* mutation [65], as well as in pathological tissues of patients with *C9ORF72*-associated ALS [66], suggesting that a subgroup of patients with familial ALS may have similar cell death cascades to those observed in most patients with sporadic ALS. Therefore, normalization of disrupted intracellular environments resulting from ADAR2 downregulation may be a therapeutic target for ALS treatment.

## 8. Conclusions and Future Directions

**Figure 8:**
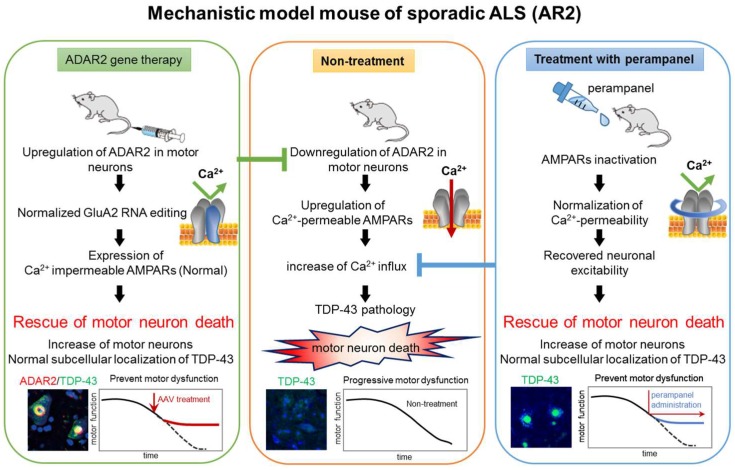
Mechanism-Based strategy for amyotrophic lateral sclerosis (ALS) therapy. Both gene therapy using adeno-associated virus as a vector (AAV9-*ADAR2* gene therapy) and treatment with perampanel significantly prevented the progression of ALS phenotypes in conditional adenosine deaminase acting on RNA 2 (ADAR2) knockout mice (AR2) by preventing transactive response DNA-binding protein (TDP-43) pathology-associated motor neuron death. Since perampanel has already been approved as an anti-epileptic drug, robust beneficial effects on the AR2 mice in these studies suggest that perampanel, ideally in combination with *ADAR2* gene therapy, would be a promising therapy for ALS. AMPAR = α-amino-3-hydroxy-5-methyl-4-isoxazole propionic acid receptor.

This review summarizes the evidence for a role of inefficient RNA editing in the pathogenesis of sporadic ALS. Inefficient RNA editing at the GluA2 Q/R site produces motor neuron death and TDP-43 pathology in motor neurons of conditional ADAR2 knockout mice, a mechanistic model of sporadic ALS, and hence likely has a pathogenic role in ALS. Furthermore, treatments including *ADAR2* gene therapy and administration of an AMPA receptor antagonist reinstated the ALS phenotype, with fragmentation and mislocalization of TDP-43 observed in the motor neurons of AR2 mice (Figure 8). We predict that upregulation of the *ADAR2* gene and perampanel administration will have synergistic effects because these therapies target different molecular abnormalities in the cell death cascade. Furthermore, both subcellular localization of TDP-43 and the number and size of ChAT-positive AHCs are useful biomarkers for the severity of ALS pathology in the mouse model. Elucidation of currently unresolved issues, such as why ADAR2 is downregulated in ALS motor neurons and how ADAR2 downregulation leads to motor neuron death, would facilitate the development of additional novel therapeutic targets.
